# Persistence of Cortical Sensory Processing during Absence Seizures in Human and an Animal Model: Evidence from EEG and Intracellular Recordings

**DOI:** 10.1371/journal.pone.0058180

**Published:** 2013-03-04

**Authors:** Mathilde Chipaux, Laurent Vercueil, Anna Kaminska, Séverine Mahon, Stéphane Charpier

**Affiliations:** 1 Centre de Recherche de l'Institut du Cerveau et de la Moelle épinière, UPMC/INSERM UMR-S 975; CNRS UMR 7225, Hôpital Pitié-Salpêtrière, Paris, France; 2 Pediatric Neurosurgery Unit, Fondation Ophtalmologique A. de Rothschild, Paris, France; 3 Grenoble Institute of Neurosciences, Centre de Recherche INSERM U 836-UJF-CEA-CHU, Equipe 9, Grenoble, France; 4 AP-HP, Service d'explorations fonctionnelles, laboratoire de neurophysiologie clinique, Hôpital Necker Enfants Malades, Paris, France; 5 UPMC University Paris 06, Paris, France; CNRS - Université Aix Marseille, France

## Abstract

Absence seizures are caused by brief periods of abnormal synchronized oscillations in the thalamocortical loops, resulting in widespread spike-and-wave discharges (SWDs) in the electroencephalogram (EEG). SWDs are concomitant with a complete or partial impairment of consciousness, notably expressed by an interruption of ongoing behaviour together with a lack of conscious perception of external stimuli. It is largely considered that the paroxysmal synchronizations during the epileptic episode transiently render the thalamocortical system incapable of transmitting primary sensory information to the cortex. Here, we examined in young patients and in the Genetic Absence Epilepsy Rats from Strasbourg (GAERS), a well-established genetic model of absence epilepsy, how sensory inputs are processed in the related cortical areas during SWDs. In epileptic patients, visual event-related potentials (ERPs) were still present in the occipital EEG when the stimuli were delivered during seizures, with a significant increase in amplitude compared to interictal periods and a decrease in latency compared to that measured from non-epileptic subjects. Using simultaneous *in vivo* EEG and intracellular recordings from the primary somatosensory cortex of GAERS and non-epileptic rats, we found that ERPs and firing responses of related pyramidal neurons to whisker deflection were not significantly modified during SWDs. However, the intracellular subthreshold synaptic responses in somatosensory cortical neurons during seizures had larger amplitude compared to quiescent situations. These convergent findings from human patients and a rodent genetic model show the persistence of cortical responses to sensory stimulations during SWDs, indicating that the brain can still process external stimuli during absence seizures. They also demonstrate that the disruption of conscious perception during absences is not due to an obliteration of information transfer in the thalamocortical system. The possible mechanisms rendering the cortical operation ineffective for conscious perception are discussed, but their definite elucidation will require further investigations.

## Introduction

**Figure 1:**
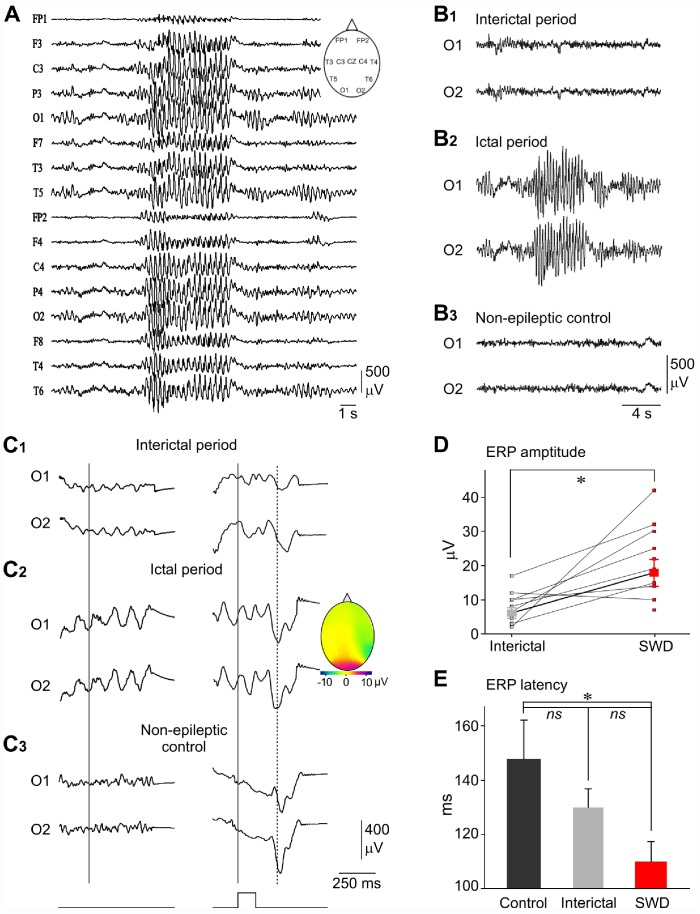
Visually-evoked ERPs are still present during cortical paroxysms in patients with absence epilepsy. (A) Bilateral spike-and-wave activity recorded from surface EEG electrodes in a 12 years-old patient with typical childhood absence epilepsy. The conventional locations of electrodes are indicated in the *inset*. (B1–B3) Typical examples of bilateral occipital EEG recordings obtained from a 12 years-old epileptic girl, between (B1) and during (B2) seizures, and from a non-epileptic subject (B3). (C1–C3) Averaging (n >90) of bilateral occipital EEG signals, during interictal (C1) and ictal (C2) periods (same patient as in B), and during a baseline period from a control patient (C3). The averaging was triggered (vertical gray line) randomly (left column) or by the light flashes (bottom traces) (right column). The vertical dashed line indicates the peak latency of the averaged visually-evoked ERPs obtained during seizures. The colour-coded topography of the ERPs amplitude (*Inset*) shows the occipital dominance of the responses. (D) Population data showing the change in ERPs amplitude evoked during interictal periods and SWDs. The lines connect the values obtained from individual patients (n = 12). In one patient, sensory responses were detectable only during seizures. The thick line indicates the mean values (± SEM) calculated from the overall population. (E) Pool data showing that the latency of sensory cortical responses was significantly reduced during SWDs (n = 13 patients) compared to that measured from control subjects (n = 7). Bar graphs represent the mean ± SEM (see results for detailed quantifications and statistical comparisons). *p<0.05; ns, nonsignificant.

The cardinal clinical symptom of absence epilepsy, which mostly occurs during childhood, is a transient impairment of consciousness [1]–[4]. These episodes of altered consciousness are temporally correlated with cycles of abnormal synchronous neural activity that involve the thalamocortical loops of the two hemispheres, resulting in bilateral 3–4 Hz spike-and-wave discharges (SWDs) in the cortical and thalamic electroencephalograms (EEGs) [3], [5], [6] (see **Figure 1A**). Although absence seizures are still classified as generalized seizures [7], EEG and magnetoencephalographic investigations from young patients demonstrated that SWDs are predominant over the frontal cortex during the epileptic episode [8], [9] and the involvement of specific left and/or right frontal regions at the onset of cortical paroxysms [10]–[12]. Consistently, a cortical focal initiation of SWDs has been found in rodent genetic models, such as the Genetic Absence Epilepsy Rats from Strasbourg (GAERS) and the WAG/Rij rats, which closely phenocopy the human absence seizures [13], [14]. In these two rat strains, SWDs are initiated from the facial somatosensory cortex [15]–[19], due to the hyperactivity and early paroxysmal discharges of the deep-layer pyramidal neurons [17], [19], [20]. These congruent findings in human and animal models led to the assumption that SWDs are generated within discrete cortical areas, and then rapidly propagate through the cortical networks and cortico-thalamo-cortical loops, allowing the maintenance of synchronized oscillations between cortex and thalamus during the seizure [16], [21], [22].

During a typical human absence, the disruption of conscious processes takes the form of a sudden arrest of ongoing behavior together with staring and unresponsiveness to environmental demand, with no recall afterwards of the epileptic attack [1]–[4]. However, the degree of consciousness impairment is highly variable between patients and even from one seizure to the next for the same subject, depending notably upon the duration of the epileptic episode and the amplitude of EEG paroxysms [2], [23], [24]. The alteration of both the level of general awareness and of sensory, or internally, generated subjective conscious events could originate from various and non-exclusive neurophysiological defects. These defects include a loss of attention mechanisms, an altered sensory integration or a global inability of cortical and subcortical networks to process information and produce conscious experience [2], [4], [25], [26]. Pioneering studies during the 1930s have shown that patients do not respond to mild visual or auditive stimuli, which will remain unremembered, whereas loud noises or pain stimuli abolish the SWD simultaneously with a recovery of consciousness [23], [27]. More recently, it has been found that patients with absence epilepsy display severe deficiency on tests of visual or auditive sustained attention compared to healthy or complex focal seizures patients [24], [28]–[30]. The break-up in visually-guided behaviors was correlated with a complete loss or pronounced attenuation of visually-evoked cortical potentials when recorded during [31] or in between [30] SWDs. These correlated behavioral and electrophysiological defects suggested the implication of two central mechanisms that could coexist or act separately. Indeed, the attenuated responsiveness during an absence could be due to an altered sensory processing and/or to a temporary dysfunction in the central attentional mechanisms [24], [30]. Alternatively, recent combined EEG and functional magnetic resonance imaging (fMRI) studies suggested that loss of consciousness during absence seizures was rather caused by a disruption of the normal information processing in specific bilateral association cortices and related subcortical structures [2], [4], [26], [32] (see Discussion).

Understanding the cerebral mechanisms of the alteration of consciousness during absence seizures also requires electrophysiological investigations at single neuron level, which are currently unconceivable in human patients. Rodent genetic models of absence epilepsy, which display strong homology with the human disease, provide a powerful experimental tool to bridge this gap by offering the possibility to analyze the neuronal and network events associated with the occurrence of absence seizures [13], [14], [22], [33]. In addition to spontaneously recurrent and bilateral SWDs in the cortex and thalamus, which are sensitive to anti-absence treatments, these rat strains exhibit during electrical paroxysms behavioral abnormalities that closely resemble the human absence [13], [14], [34]. In the GAERS, SWDs, which start and end abruptly on a normal background EEG, are concomitant with behavioral immobility and rhythmic twitching of the vibrissae and facial muscles [13]. As in patients with typical absence epilepsy, GAERS do not display other neurological defects and do not show any modification in their spontaneous motor activity, exploration, feeding or social interactions, compared to non-epileptic rats [13], [35]. As in human patients, the responsiveness to mild sensory stimuli is abolished during SWDs and the performance on instrumental learning tasks is considerably impaired when the conditioning sensory stimulus coincides with the occurrence of cortical paroxysms [13], [35], [36].

In the sole investigation specifically designed to determine whether external sensory inputs can be processed in the neocortex during human absences, visual evoked cortical potentials during seizures were found dramatically degraded or absent. This apparent deficiency of cortical responsiveness was possibly due to the small number (n = 3) of tested subjects and/or an insufficient number of averaged responses, without comparison with control subjects [31]. Here, we first quantify, in young patients with drug-resistant typical absence and in the GAERS, the properties of surface event-related potentials (ERPs) recorded from the cortical areas related to the stimulated sensory channels. In epileptic patients and healthy human subjects, intermittent flash light stimulations [37], [38] were applied and occipital ERPs were averaged and measured. In GAERS, we further examined the sensory integration during SWDs by making combined EEG and intracellular recordings from the facial part of the somatosensory cortex and analyzing the surface and intracellular responses to air-puff stimulations applied to the contralateral whiskers [39]. In both species, ERPs and/or intracellular responses evoked during SWDs were compared to those obtained during interictal periods and during normal cortical activities from non-epileptic subjects.

## Materials and Methods

### Human Recordings

This study followed the principles of Declaration of Helsinki for human subject protection, and the protocols were approved by the local Ethical Committee (CPP-Ile-de-France VI). Written informed consent from the parents and written assent from the children were acquired for each participant.

#### Patients

We recorded 13 children with drug-resistant typical childhood absence-epilepsy, in accordance with the International League Against Epilepsy criteria [40], and 7 healthy control children. Epileptic and control children were studied in four neuropediatrician units in France: Necker Enfants-Malades, Paris; Rothschild Foundation, Paris; Grenoble University Hospital and Lille University Hospital. Children were video monitored, notably to ensure that SWDs were concomitant with absences. Exclusion criteria were visual disorders, other epilepsies than childhood absence-epilepsy, neurological disease, mental retardation, abnormal brain imaging and history of photically induced seizures.

#### EEG recordings

Each EEG recording, lasting at least 30 min, contained 2 periods (≥3 minutes) of hyperventilation in order to trigger absences in epileptic children [41]. Twenty-one scalp electrodes were positioned according to the classical 10–20 system [42]. The EEG was recorded with an average reference. Signals were amplified (1000 times), filtered at 0.05 to 97 Hz and acquired at 256 Hz using the Deltamed Natus (San Carlo, USA) or Micromed system (Mogliano Veneto, Italy).

#### Sensory stimulations

We chose to apply photic stimulations because the visual system provides the main sensory channel involved in perceptual consciousness in human [43]. Stimulations were given with a standard photostimulator positioned at a distance of 25 cm from the eyes. Direct flashes (800 lux during 100 ms) were automatically applied at 2 Hz during 2 hyperpnoea periods lasting at least 3 minutes. The 2-Hz frequency was chosen to allow an analysis period of 300 ms after each stimulation, including the latency of the cortical visually-evoked ERP (vERP). At least 30 stimuli were delivered during interictal periods and baseline activity in healthy subjects.

#### Data analysis

EEG segments with visual stimulation were manually selected to discard the periods contaminated by muscular artifacts. Averaging of successive individual responses was performed for each electrode with mean reference, during both ictal and interictal periods. We focused our analysis on occipital (O1 and O2) records because vERPs with maximal amplitude were located at occipital sites [37], [38] (**Figure 1C** **_2_, inset**). According to the classical procedures [30], [38], latency of vERPs was calculated as the time between the onset of the flash and the peak of the sensory response, *i.e.* at a time where the signal-to-noise ratio is maximal. Random averaging (n = 100 trials) were made from interictal and ictal periods in epileptic children, and from control activity in non-epileptic subjects, to assess the possible participation of spontaneous EEG waves in the averaged sensory-evoked responses. The color-coded spatial representation of the maximum amplitude of vERPs was performed using Micromed software (Mogliano Veneto, Italy).

### Animal Experiments

The care and experimental manipulation of the animals followed the European Union guidelines (directive 86/609/EEC). Protocols strictly conformed to the regulation of the Bureau d’Expérimentation Animale of the Institut National de la Santé Et de la Recherche Médicale (Licence B-75-1136) and were approved by the Office of Laboratory Animal Welfare (Assurance Number A5326-01). Every precaution was taken to minimize suffering (see anesthesia procedures below) and the number of animals used in each series of experiments.

#### Animal preparation for in vivo EEG and intracellular recordings

Electrophysiological recordings were performed *in vivo* from three- to ten-month-old GAERS (n = 11) and non-epileptic adult Wistar rats (n = 11) (Charles River, L’Arbresle, France). Detailed procedures of anesthesia and surgery are described in details elsewhere [17], [19], [20]. Briefly, anesthesia was first induced with intraperitoneal (i.p.) injection of sodium pentobarbital (40 mg/kg body weight) and ketamine (50 mg/kg body weight) for surgery procedures. Animals were then artificially ventilated after immobilization by gallamine triethiodide (40 mg i.m. every 2 h). Sedation and analgesia was maintained throughout the recording sessions by repeated injections of fentanyl (3 µg/kg body weight, i.p.) allowing the spontaneous occurrence of SWDs on a waking-like background EEG activity [17], [19], [44]. Body temperature was maintained (36.5–37.5°C) with a homoeothermic blanket. At the end of the experiments, animals received an overdose of sodium pentobarbital (200 mg/kg body weight, i.p.).

#### Electrophysiological recordings

**Figure 2:**
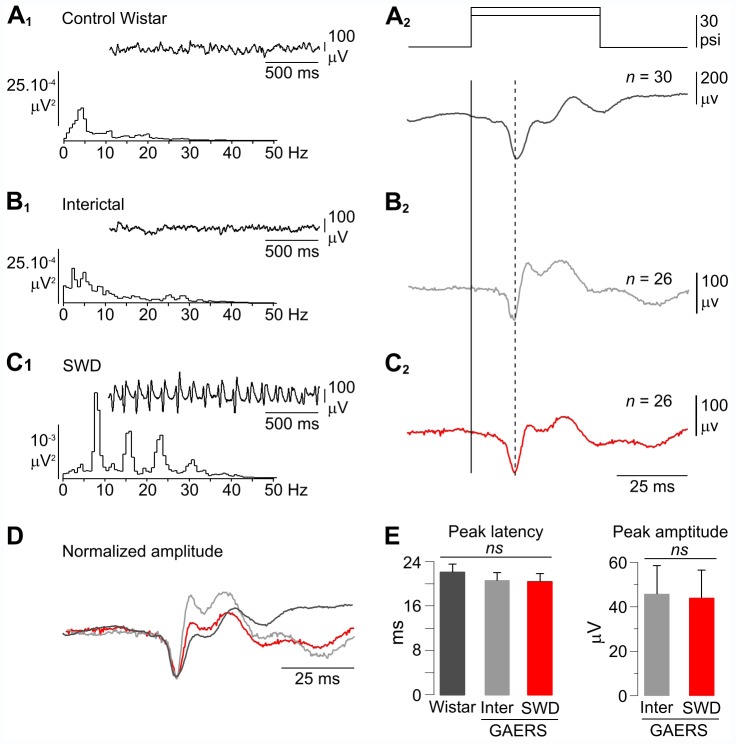
wERPs are not significantly altered during SWDs in the GAERS. (A1–C1) Frequency power spectra computed from 5 s of spontaneous EEG activity, including the truncated records shown in the corresponding *insets*, from a non-epileptic rat (A1) and during interictal (B1) and ictal (C1) periods in a GAERS. (A2–C2) Average (the number of trials is indicated) wERPs obtained in the three conditions as shown in A1–C1, in response to air-puff stimuli applied to the contralateral whiskers (top traces). The latency of wERPs was measured as the time difference between the onset of the air-puff (solid line) and the peak of the first negativity of evoked potentials (dashed line). (D) Superimposition of the average records shown in A2–C2, with normalized amplitude (using the initial negative component as the amplitude reference) and using the onset of the sensory stimulus as the time reference. Note the constancy in latency and shape of the first component of the wERPs in the non-epileptic Wistar rat and the GAERS. (E) Pooled data showing that the peak latency of the early sensory responses was not significantly different in the three conditions (Control Wistar rats, n = 11; Interictal periods (Inter) and SWDs in GAERS, n = 11; p = 0.4) (left), with an amplitude during SWDs that remained unchanged compared to the corresponding interictal periods (n = 11 GAERS; p = 0.7) (right). Bar graphs represent the mean ± SEM (see results for detailed quantifications).

**Figure 3:**
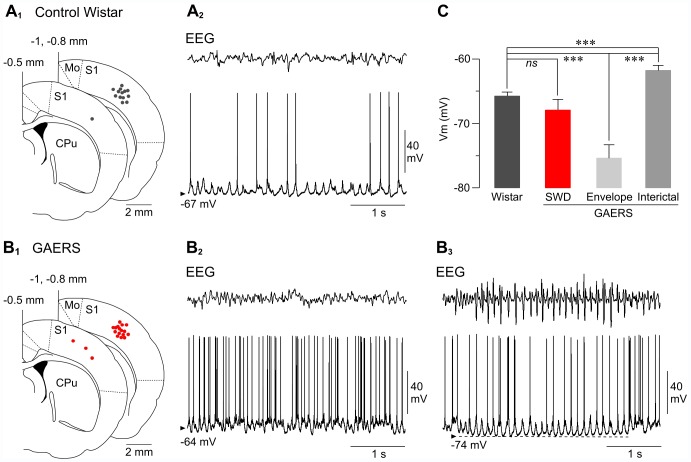
Spontaneous intracellular activities of S1 cortex neurons from non-epileptic rats and GAERS. (A1–B1) Superimposed slice drawings, made from the stereotaxic rat brain atlas from Paxinos and Watson (1986) at the indicated distances (in millimetres) from the bregma. Black and red dots indicate the location of intracellularly recorded neurons from the S1 cortex of control Wistar rats (A1) and GAERS (B1), respectively. Mo, motor cortex; CPu, caudate-putamen. (A2, B2, B3) Simultaneous recordings of spontaneous intracellular activities (bottom records) and corresponding EEG waves (top records) from a non-epileptic rat (A2) and from a GAERS, during interictal (B2) and ictal (B3) periods. Note that the interictal irregular membrane potential fluctuations and firing pattern was replaced, at the occurrence of a SWD, by rhythmic suprathreshold membrane depolarizations superimposed on a tonic hyperpolarization that lasted for the entire epileptic episode. The arrowheads indicate membrane potential values. Records shown in B2 and B3 are from the same neuron. (C) Pooled values of mean membrane potential (Vm) from pyramidal neurons recorded in normal rats (Wistar, n = 14 neurons) and in GAERS (n = 19 neurons) during SWDs, at the sustained hyperpolarization associated with seizures (Envelope) and during interictal periods (Interictal). ***p<0.001; ns, nonsignificant.

Spontaneous EEG activity and surface ERPs (**Figure 2A–D**) evoked by contralateral stimulations of whiskers (wERPs) were recorded with a low impedance (∼60 kΩ) silver electrode placed on the dura above the facial region of the primary somatosensory (S1) cortex [45] at the following coordinates: 0 to −1.5 mm posterior to the bregma and from 4 to 5.5 mm lateral to the midline. The reference electrode was placed in a muscle on the opposite side of the head. Intracellular recordings were performed using glass micropipettes filled with 2 M potassium acetate (50–80 MΩ). Neurons recorded from GAERS and control Wistar rats were located within the same region of the S1 cortex (**Figure 3A** **_1_ and 3B_1_**) and close to (<500 µm) the surface EEG electrode, at the following stereotaxic coordinates: −0.5 to −1.0 mm posterior to the bregma, 3.8–5.5 mm lateral to the midline, and 904–2713 µm under the cortical surface. These anatomical coordinates indicated that intracellularly recorded cells were located in the deep layers of the S1 cortex area previously identified as the cortical trigger for absence seizures in the GAERS [17], [19].

#### Sensory stimulations

Sensory stimulations, consisting in puffs of compressed air delivered by a pressure device (Picospritzer III, Intracel Ltd, Royston, Herts, UK), were applied through a 1 mm diameter glass pipette placed 15–25 mm rostrolateral from the whiskers. Square air puffs (50 ms duration) were given 20–100 times for each intensity tested (ranging between 10 and 50 p.s.i.) with a low frequency (0.24 Hz) to prevent adaptation of whisker-evoked responses [46]. To closely replicate natural sensory stimulations, multi-whisker stimulations were preferred to single whisker stimulations as they are more likely to occur during exploratory behavior [47], [48]. The intensity of optimal sensory stimulus was determined, in absence of epileptic discharge, as the minimal air-puff pressure generating a contralateral ERP of maximal amplitude (40–60 µV). Under these conditions, the air-puff stimuli deflected 4–8 whiskers by ∼10 deg.

#### Data acquisition and analysis

Electrophysiological signals were digitized and stored on-line with a sampling rate of 25 kHz (intracellular recordings) or 3 kHz (EEG, ERP). Intracellular recordings were obtained under current-clamp conditions using the active bridge mode of an Axoclamp-2B amplifier (Molecular Devices, Union City CA, USA). The start and end of a SWD in the GAERS EEG were taken to be the first and last spike component, respectively, where the size of the spike was at least two times the peak-to-peak amplitude of the baseline EEG. To perform spectral analysis of EEG potentials (over a period of at least 5 s), fast Fourier transforms were applied using Spike2 software (Cambridge Electronic Design, Cambridge). The value of neuronal membrane potential was calculated as the mean of the distribution of spontaneous subthreshold activity (>10 s duration). The membrane potential values were eventually corrected when a tip potential was recorded after termination of the intracellular recording. Apparent membrane input resistance of cortical neurons was measured by the mean (n = 20) membrane potential change at the end of hyperpolarizing current pulses of −0.4 nA. The membrane time constant was derived from an exponential decay fit applied to the current-induced hyperpolarization. Neurons with action potentials having an amplitude <50 mV were not included in the database. Voltage threshold of action potentials was measured as the membrane potential at which the dV/dt first exceeded 10 V.s−1 [49]. In GAERS and control Wistar rats, the latency of wERPs, in presence and in absence of epileptic discharges, was measured as the time between the onset of the air-puff stimulus and the peak of the first negative component of the surface cortical response, which reflects the initial synaptic population response in the underlying cortical network [39], [50]. The latency of the corresponding intracellular responses was calculated as the time between the onset of the air-puff stimulus and the foot of the evoked potential. Neuronal events having a shape (rising and decay phases) and/or latency that did not match those of the mean synaptic response obtained after averaging of all trials applied in the same condition (with or without SWDs) were not considered as induced by the whisker stimulation and discarded [39]. The amplitude of individual sensory-evoked subthreshold potentials was measured as the voltage difference between the membrane potential at the foot and the peak of the response. Latency of evoked spikes on suprathreshold synaptic responses was the time between the start of the sensory stimulus and the peak of the spike waveform. The firing probability of neurons in response to whisker deflections was calculated as the ratio between the number of suprathreshold synaptic responses and the total number of sensory-evoked responses.

### Statistical Analysis

Numerical values are given as mean ± standard error of the mean (SEM). Statistical significances were assessed using unpaired two-tailed Student’s t tests, one-way ANOVA, or the non-parametric Mann-Whitney rank sum test and Kruskal-Wallis ANOVA on ranks. Statistical analyses were performed with SigmaStat 3.1 (SPSS Inc., Chicago, IL, USA).

## Results

### Persistence of Visually-induced ERPs in Human Patients with Absence Epilepsy

We examined the neocortical sensory responses in 13 children with drug-resistant typical childhood absence epilepsy and 7 non-epileptic children, using multi-site EEGs (**Figure 1A**) and intermittent flash light stimulations (see **Materials and Methods**). Epileptic and control healthy children had approximately the same age (epileptic children, 11.9±1.1 years old, 6–18 years, n = 13; control children, 11.6±1.7 years old, 6–18 years, n = 7). With respect to antiepileptic medications, the most common was lamotrigine, followed by levetiracetam and sodium valproate. Three epileptic children received a combination of two antiepileptic drugs and three others were off treatment because no medication was effective. A total of 153 absences were recorded, including 45 episodes during which visual stimulations were given. Epileptic paroxysms could be detected from all EEG electrodes (**Figure 1A**), including the occipital derivations (**Figure 1B** **_2_**). They had a duration ranging from 1 to 50 seconds, an optimal internal frequency between 3 and 3.5 Hz and displayed variable amplitude between patients.

In nearly all children and recording conditions, including quiescent periods in healthy subjects, interictal and ictal periods from epileptic subjects, light flashes evoked detectable vERPs after averaging (>30 successive trials) of EEG waves recorded from the occipital electrodes (**Figure 1C** **, right**). However, in one patient, although no vERPs could be detected during interictal epochs, despite the large number of trials (n = 61), a clear average cortical response was evidenced during epileptic episodes. The reverse situation was never observed. The random averaging of EEG records during the three conditions did not produce any wave having the latency and the shape of those evoked by the flashes (**Figure 1C** **, left**). This indicates that the sensory responses recorded during absences were not significantly contaminated by the spontaneously occurring spike-and-wave complexes.

When sensory stimulations were delivered during seizures, the amplitude of occipital vERPS was mostly enhanced (n = 10 out 13 epileptic children) compared to interictal periods in the same patient (ictal vERPs, 18.0±3.6 µV, n = 13 patients; interictal vERPs, 6.2±1.5 µV, n = 13 patients, p = 0.02) (**Figure 1C** **_2,3_ right and 1D**). We considered amplitude comparison between different children unreliable due to differences in skull and electrode impedances, which may alter the absolute amplitude of inter-individual EEG signals. During seizures, vERPs occurred more promptly, with a significant decrease in their latencies (109.6±7.3 ms, n = 13 patients) compared to that measured from control children (147.7±14.4 ms, n = 7 subjects, p = 0.025) (**Figure 1C** **right and 1E**). However, the peak latency of the sensory response during absences was slightly, but not significantly, shorter compared to the corresponding interictal periods (129.83±6.99 ms, n = 12 patients, p = 0.06) (**Figure 1C** **right and 1E**). No significant difference was found between the latencies of vERPs generated during interictal epochs and baseline activity in healthy subjects (p = 0.4). There were no correlative relationships between vERPs amplitude and latency and the anti-absence drugs administrated.

All epileptic patients were completely unresponsive during seizures, with no post-ictal recollection of the sensory stimuli applied during absences.

### Whisker-evoked ERPs are not Altered during SWDs in the GAERS

Our recordings from epileptic children indicated that visual stimuli applied during seizures, although not consciously detected, could access and be processed by the cerebral cortex, as evidenced by the reproducible vERPs at occipital regions, which could be even enhanced in amplitude compared to that recorded during interictal periods. We next tested whether sensory events can be processed in the cerebral cortex of the rat genetic model during SWDs, using a sensory channel, the vibrissae system, essential for tactile perception of the environment in rodents [48]. We thus compared the whisker-evoked responses recorded in the S1 cortex during interictal periods and SWDs in GAERS (n = 11) to those obtained from the same cortical region in control Wistar rats (n = 11).

Background EEG activity recorded under fentanyl sedation from the S1 cortex of control rats (**Figure 2A** **_1_**, **top record**) and GAERS between seizures (**Figure 2B** **_1_**, **top record**) had a low-amplitude desynchronized pattern and displayed similar frequency contents, with a preferred frequency at 2–4 Hz intermingled with faster activity up to 40 Hz (**Figure 2A** **_1_ and 2B_1_**). At the occurrence of seizure activity, the interictal desynchronized cortical EEG of GAERS was replaced by large-amplitude SWDs (**Figure 2C** **_1_ and 3B_3_, top record**), having an internal frequency of 7.9±0.2 Hz, a mean duration of 5.7±2.3 s and spontaneously recurring with highly variable time intervals (95±67 s, n = 416 SWDs from 11 GAERS) (**Figure 2C** **_1_ and 3B_3_, top record**). The overall properties of SWDs recorded in this study are similar to those described previously under analogous experimental conditions [17], [19], [20], [34] and in freely moving GAERS [13], [17].

**Figure 4:**
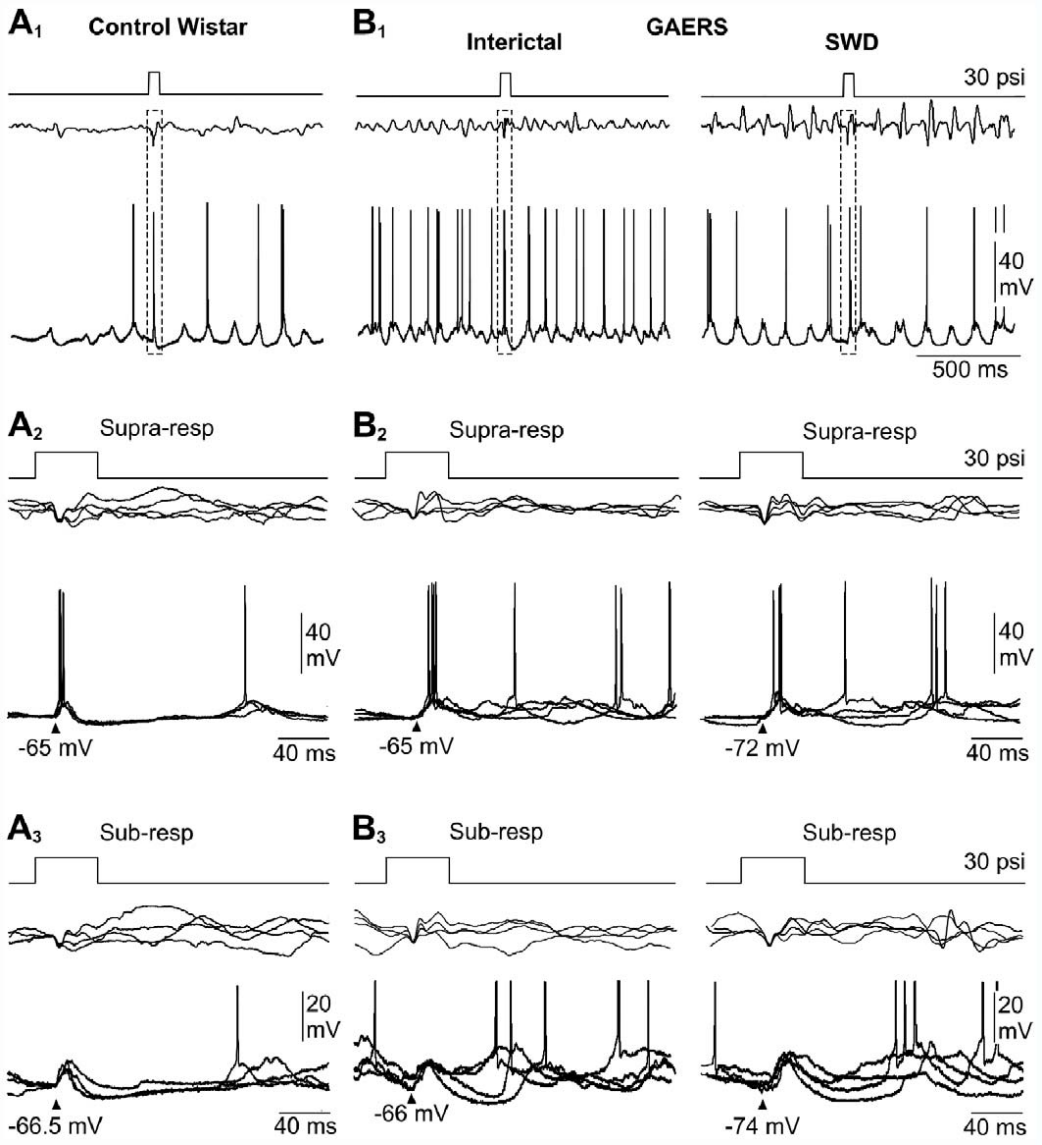
Sensory responses intracellularly recorded in S1 cortex neurons from non-epileptic Wistar rats and GAERS. Each panel depicts the sensory stimulus (top traces) and the corresponding responses in the EEG (middle records) and in a pyramidal neuron (bottom records) simultaneously recorded. (A1, B1) Typical examples of single sensory-evoked responses recorded from a control Wistar rat (A1) and in a GAERS (B1), during an interictal epoch (left) and during a seizure (right). The dashed boxes enclose the responses induced by whiskers deflection. (A2, B2) Superimposition (n = 4) of wERPs and corresponding suprathreshold intracellular responses (Supra-resp) in control (A2) and epileptic (B2) animals, during interictal (B2, left) and seizure (B2, right) periods. (A3, B3) Same representation as in A2 and B2 for neuronal responses that remained subthreshold in the three conditions. In A2, A3, B2, B3, the mean value of membrane potential at the onset of the cellular responses is indicated by the arrowheads. In A3 and B3, action potentials are truncated for clarity. Results depicted in A and B are from single neurons.

Square air-puff stimuli were repeatedly applied on whiskers of non-epileptic rats and GAERS, in between and during seizures, with the minimal intensity to produce the largest wERP in absence of cortical paroxysmal activities (10–40 p.s.i., interictal period in GAERS; 10–50 p.s.i., control Wistar rats). Using this range of intensities, whisker stimulations did not interrupt the seizures (see **Figure 4B** **_1_ right**) and the stimulus-trigger averaging (>20 trials) of cortical EEG waves revealed a clear-cut wERP during SWDs (**Figure 2C** **_2_**). In most cases, individual sensory-evoked responses could be identified and distinguished from the spike component of the spike-and-wave complex due to their reproducible shapes and latencies (see **Figure 4B** **_2,3_, top records at right**). wERPs recorded concurrently with SWDs had similar complex waveforms than those acquired during interictal epochs and from control rats, including a consistent early negative component of large amplitude followed by 2 to 3 deflections with variable amplitude and of positive or negative polarity (**Figure 2A** **_2_, 2B_2_, 2C_2_ and 2D**) [39], [50], [51]. The peak latency of the early component of the wERPs, calculated after averaging of 20 to 40 successive trials, was similar in the three conditions (control Wistar rats, 22.0±1.5 ms, n = 11 animals; GAERS interictal, 20.5±1.5 ms; GAERS ictal, 20.4±1.5 ms, n = 11 animals; p = 0.4) (**Figure 2D and 2E** **, left**). Moreover, we did not measure any significant modifications in the average amplitude of the early wERPs wave recorded during cortical paroxysms and interictal periods (GAERS ictal, 45.7±12.7 µV; GAERS interictal 44.0±12.4 µV, n = 11 animals; p = 0.7) (**Figure 2B** **_2_, 2C_2_ and 2E, right**).

### Spontaneous Activity and Excitability of S1 Cortex Neurons in GAERS and Control Wistar Rats

The stability of latency of wERPs in non-epileptic rats compared to GAERS, and the constancy of their amplitude during SWDs compared to interictal conditions, confirm and extend our findings from human patients that absence seizures do not preclude the conveying of sensory inputs towards the cerebral cortex and their processing by cortical networks. However, these surface field potentials do not allow determining whether the synaptic responses and output activity of individual cortical neurons are altered during seizures. Thus, we made concomitant EEG and intracellular recordings from quiescent periods in control Wistar rats and during seizures and interictal epochs in GAERS, and compared the cell responses between the three conditions.

Intracellular recordings from non-epileptic rats (n = 14 neurons from 11 rats) and GAERS (n = 19 neurons from 10 GAERS) were performed in the facial part of S1 cortex, previously identified as the cortical area generating SWDs in rodent genetic models [15], [17], [19], [52]. In control rats (**Figure 3A** **_1_**) and GAERS (**Figure 3B** **_1_**), recorded cells were located at similar coordinates and within layers 5 and 6 (see **Materials and Methods**). Their current-evoked firing patterns were mostly regular spiking (7 out of 14 neurons in Wistar rats; 14 out 19 neurons in GAERS) or intrinsic bursting, indicating that neurons were of pyramidal type [53]. Pyramidal cells of non-epileptic rats displayed spontaneous depolarizing synaptic events of variable amplitude, leading to a mean membrane potential of −65.7±0.6 mV (n = 14 neurons) (**Figure 3A** **_2_ and 3C**) and responsible, in the active cells (n = 11 out 14 neurons), for a moderate background firing rate (3.1±0.8 Hz, n = 11) (**Figure 3A** **_2_ and 3C**). As previously described [17], [18], [34], S1 cortical neurons of GAERS displayed during interictal periods a frequency of spontaneous discharge (6.7±1.1 Hz, n = 19 neurons) (**Figure 3B** **_2_**) significantly more elevated (p<0.01) than that of homotypic non-epileptic neurons, due to a more depolarized membrane potential (−61.8±0.8 mV, n = 19 neurons, p<0.001, **Figure 3C**). The occurrence of a SWD in the EEG was accompanied in GAERS cortical neurons with suprathreshold oscillatory membrane depolarizations that were temporally correlated with the surface cortical oscillations (**Figure 3B** **_3_**). These repetitive membrane depolarizations were superimposed on a tonic hyperpolarization of 12.5±1.61 mV (n = 416 SWDs from 19 neurons), lasting for the entire epileptic activity and reaching a mean value of −75.4±2.1 mV (n = 416 SWDs from 19 neurons) (**Figure 3B** **_3_ and 3C, envelope**), which was significantly more negative compared to that measured from interictal periods and from baseline activity in non-epileptic animals (p<0.001) (**Figure 3C**). The mean membrane potential during seizures (−67.9±1.7 mV, n = 416 SWDs from 19 neurons), including oscillatory depolarizations and excluding action potentials, was similar to that of non-epileptic rats (p = 0.1) (**Figure 3C**).

Neurons recorded from non-epileptic rats and GAERS did not show any significantly differences in other basic electrophysiological features, including action potential threshold (Control Wistar rats, −51.5±0.9 mV, n = 14 neurons; GAERS, −51.1±0.7 mV, n = 19 neurons; p = 0.7) and membrane time constant (control Wistars, 8.1±0.4 ms, n = 14 neurons; GAERS, 8.6±0.5 ms, n = 18 neurons; p = 0.5). Only the apparent membrane input resistance of GAERS neurons (28.6±2.1 MΩ, n = 18 neurons), measured during interictal periods, was enhanced compared to that calculated in normal rats (20.6±1.1 MΩ, n = 14 neurons; p = 0.01).

### Intracellular Responses of S1 Cortex Neurons to Sensory Inputs during Quiescent Periods and SWDs

To determine whether sensory integration is altered in individual cortical neurons, we next compared the intracellular responses of S1 cortex neurons of non-epileptic (n = 14 neurons from 11 rats) and epileptic rats (n = 11 neurons from 8 GAERS) to air-puff stimuli applied to the contralateral whiskers (**Figure 4A** **_1_ and 4B_1_**). In each experiment, the pressure intensity was preliminarily adjusted, in absence of cortical paroxysms, to produce an optimal wERP (see **Materials and Methods**).

In non-epileptic rats, low-frequency (0.24 Hz) mechanical stimulations of whiskers generated in S1 cortex neurons sub- or suprathreshold depolarizing postsynaptic potentials (dPSPs) having a mean latency, relative to the onset of the stimulus, of 17.6±0.5 ms (n = 349 responses from 14 neurons) (**Figure 4A** **and** **5A**). In 9 out of 14 tested cells (∼64%), sensory-evoked responses could evoke an action potential with a mean probability of 0.42±0.1 (n = 9) (**Figure 4A** **_2_ and 5C**) and an average latency of 23.6±1.9 ms (n = 9) (**Figure 4A** **_2_ and 5D**). The subthreshold responses had a mean amplitude of 6.8±0.6 mV (n = 14 neurons) (**Figure 4A** **_3_ and 5B**).

**Figure 5:**
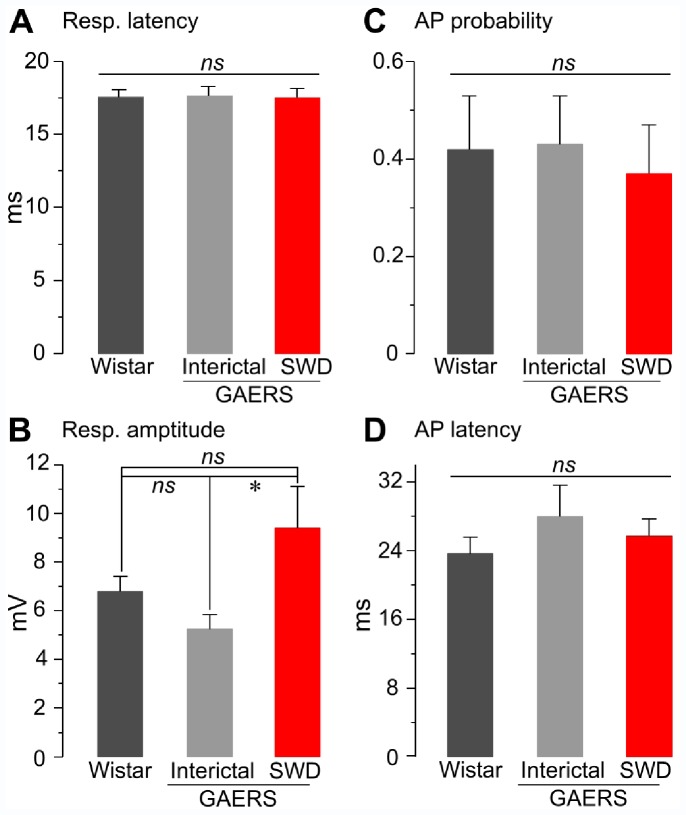
Properties of sensory-evoked intracellular responses from non-epileptic rats and GAERS. Pooled data of the mean latency of sensory-evoked responses (A, Resp. latency), mean amplitude of subthreshold dPSPs (B, Resp. amplitude), action potential firing probability (C, AP probability) and corresponding latency (D, AP latency) from non-epileptic Wistar rats (Wistar) and GAERS, during interictal and seizure (SWD) periods. Only the amplitude of subthreshold dPSPs during SWDs was found significantly different compared to that measured during interictal activity. *p<0.05; ns, nonsignificant. Bar graphs represent the mean ± SEM (see results for detailed quantifications).

The intracellular responses recorded from GAERS cortical neurons during interictal periods (**Figure 4B** **_1_, left**) were globally similar to that of neurons recorded from non-epileptic rats. First, the latency of sensory-evoked responses, including sub- and suprathreshold events, was 17.6±0.6 ms (n = 180 responses from 11 neurons, p>0.9). Second, the proportion of epileptic neurons that could respond by suprathreshold dPSPs was roughly the same (54%, 6 out of 11 neurons) and the probability with which individual dPSPs could generate an action potential (0.43±0.1, n = 6) was identical (p = 0.8) to that calculated from non-epileptic animals. Moreover, the corresponding latency of discharge (28.0±3.7 ms, n = 6) was also found similar compared to control neurons (p = 0.2). Finally, the amplitude of subthreshold dPSPs (5.2±0.6 mV, n = 11 neurons) (**Figure 4B** **_3_ and 5C**) was slightly, but not significantly (p = 0.08), reduced compared to responses recorded from non-epileptic animals.

One of the major outcomes of the present study was the description of the cortical intracellular responses to sensory stimuli during absence seizures. GAERS neurons could respond during seizures (**Figure 4B** **_1_, right**) by sub- or suprathreshold dPSPs, with a mean latency (17.5±0.6 ms; n = 351 responses from 10 neurons, n = 8 GAERS) (**Figure 4B** **_2_, right**) consistent with that calculated from control neurons (p>0.9) and from the same neurons during the corresponding interictal states (**Figure 5A**, p = 0.9). Moreover, the occurrence of epileptic seizures did not reduce the number of neurons that could be fired by the whisker stimulations (9 out of 10 neurons, 90%), with a probability to generate an action potential in individual neurons (0.37±0.1, n = 9) matching that measured in the two other conditions (p>0.4) (**Figure 4B** **_2_ right and 5C**). The latency of sensory-evoked action potentials during SWDs (25.7±2.0 ms, n = 9 neurons) remained also unmodified compared to control and interictal responses (p>0.4) (**Figure 4B** **_2–3_ and 5D**). The membrane potential at the occurrence of sensory-induced synaptic responses during seizures was more polarized (−70.6±2.2 mV, n = 351 responses from 10 neurons) compared to interictal conditions (p<0.05), with a value matching the mean membrane potential previously calculated during seizures (p = 0.3) (**Figure 3C** **, SWD**). The sensory-induced dPSPs that remained subthreshold for action potential discharge during seizures had a mean amplitude (9.4±1.7 mV, n = 10 neurons) significantly larger (p = 0.01) than that measured in between SWDs but close to that measured from non-epileptic rats (p = 0.1).

## Discussion

In the present study, we examined how sensory inputs, generated by natural sensory stimuli, are processed by the cerebral cortex during absence seizures. In both epileptic patients and GAERS, a well-established genetic model, cortical ERPs were still present when the stimuli were delivered during SWDs, without significant modification in shape compared to responses obtained during interictal periods and from non-epileptic subjects. Visually-evoked occipital ERPs in epileptic patients were even increased in amplitude and reduced in latency during cortical paroxysms. Using *in vivo* intracellular recordings of S1 cortex pyramidal neurons from GAERS and control rats, we found that whisker-evoked synaptic responses was increased in amplitude during the epileptic episode, without significant change in their latency and their capacity to fire action potentials. Contrasting with earlier works suggesting a complete or partial obliteration of information transfer during absences [25], [30], [31], our findings demonstrate that external inputs can still access the cerebral cortex and be processed by local networks and neurons.

### Sensory Integration in the Thalamocortical Pathway during SWDs

In both human patient and epileptic rodent, surface ERPs were preserved when sensory stimuli were delivered during SWDs. Conversely, EEG epileptic discharges were not disrupted by the occurrence of cortical sensory responses, at least with the range of stimulus intensity used in the present study. These data indicate that the cellular and network mechanisms eliciting and/or sustaining spike-and-wave activities and sensory-evoked cortical potentials are not mutually exclusive. This is consistent with recent findings demonstrating that SWDs in the GAERS are initiated from synaptic interactions between ictogenic pyramidal neurons located in layer 5–6 of the somatosensory cortex [17], [19], [20], whereas ascending sensory inputs resulting from mechanical stimulation of the whiskers travel to the brainstem, project onto excitatory thalamocortical neurons, which in turn predominantly connect layer 4 stellate cells of the S1 cortex [48], [54]. However, ictogenic and sensory cortical networks are partially interconnected and it is likely that more intense or painful stimuli will induce widespread cortical desynchronization [27], [55] including the cortical region initiating seizures, and, consequently, will lead to the interruption of paroxysmal oscillations and the recovery of conscious processes [22], [27].

It is very unlikely that fentanyl sedation used in GAERS experiments may have interfered with the whisker-induced sensory responses. Indeed, the spontaneous activity and receptive field size of neurons in the ventro-posterior medial (VPm) thalamic nucleus, which transmit whisker-dependent sensory inputs to the S1 cortex, are similar under fentanyl and in awaked animals [44]. Moreover, sensory-evoked responses in the layer 4 neurons of S1 cortex do not significantly differ between sedation and wakefulness [56].

The persistence of cortical responses when external stimuli are presented during SWDs challenges the assumption that sensory information is transmitted from the thalamus to the cortex only during the thalamic tonic firing mode, and that no information is transmitted during the oscillatory mode [21], [25], [57]–[59]. In contrast, our findings suggest that oscillatory behavior of thalamic neurons during absence seizures could be ideally tuned to detect fast changes in incoming sensory signals and to pass this information toward the cortex. During SWDs, sensory thalamocortical neurons exhibit membrane potential oscillations caused by regenerative low-threshold Ca2+ spikes, which are promoted by a prolonged membrane hyperpolarization (to around –70 mV) [19], [21], [60] due to a powerful GABAergic inhibition arising from the bursting of nucleus reticularis thalami (nRT) neurons [21], [61], [62]. At the hyperpolarizing phase of the cycle, the polarization of thalamic cells is sufficient to de-inactivate the T-type Ca2+ channels which are then ready to be activated by a sufficient depolarization, such as one generated by a sensory-evoked excitatory synaptic potential [63], leading to a Ca2+ spike and a burst of sodium action potentials that propagate to the cerebral cortex. A similar process is presumed to facilitate the detection of novel tactile stimuli during the 7–12 Hz rhythm in the somatosensory thalamocortical pathway of Long-Evans rats [54], [64], which is supposed to be a functional analog of the physiological human μ rhythm [64] or an absence-like activity [65], [66].

The sensory-induced synchronization of thalamocortical ascending activity could produce fast and coherent depolarizing synaptic depolarizations in cortical neurons, responsible for the early component of surface ERPs. The increased amplitude of subthreshold dPSPs in S1 cortex neurons of GAERS during SWDs could result from two synergistic phenomena. First, the sustained membrane hyperpolarization of cortical cells accompanying seizures could increase the driving force of excitatory synaptic currents and, thus, the amplitude of dPSPs compared to the depolarized interictal state. Second, the steady polarization of cortical neurons resulting from a synaptic disfacilitation, *i.e.* temporal absence of tonic network activity, could lead to an increase in membrane input resistance responsible for an amplification of synaptic potentials [21], [67]. Assuming the existence of a similar cellular process in human cortical cells, the enlargement of sensory-induced dPSPs in many occipital neurons during seizures may account for the increased amplitude of vERPs. The mechanisms by which vERPs are more prompted when the stimulus is presented during SWDs remain unclear. However, it is plausible that the burst-responses caused by sharp Ca2+ spikes will be rapidly and tightly synchronized among thalamocortical cells integrating the same sensory inputs [54] reducing thereby the latency of the sensory cortical responses compared to the more graded responses generated during desynchronized activity in the primary visual cortex [68].

### Sensory Responses in the Cortex Without Conscious Experience

We demonstrated that specific sensory stimuli are able to produce accurate and reliable neuronal responses in the related cortical areas during SWDs, whereas the subjects remain unresponsive. Our findings thus dispute the widely accepted assumption that synchronized oscillations in thalamocortical loops disrupt conscious perception by filtering-out external sensory inputs and/or disallowing their allocation to the appropriate cortical assemblies [21], [25], [57]–[59], [69], [70]. Nonetheless, they are in fact in agreement with a number of studies, ranging from single unit recordings in animals [71] to human EEG [72] and neuroimaging [73] experiments, which demonstrate the persistence of brain responses to sensory stimulations during non-rapid eye movement sleep, suggesting that the brain can still process external stimuli during thalamocortical oscillations that make the subject unresponsive. In addition, in patients with cortical blindness, unaware of the visual stimulus, vERPs can be recorded [74]. More recent studies combining EEG and fMRI in human indicate that auditory cortical responses persist during non-rapid eye movement sleep, except during spindles [75], [76] and the negative going phase of the slow-wave oscillations during which responses become less consistent or even absent [76]. This suggests that processing of sensory inputs during thalamocortical rhythms is strongly influenced by the phase of the oscillation at which stimuli are delivered [77].

During absence seizures, the functional dissociation between the electrophysiological responses in primary sensory cortices and the lack of conscious perception remains enigmatic. However, since the internal frequency of seizures is of ∼2–4 Hz in human and ∼7 Hz in the GAERS, any cortical response to external stimuli will be necessarily concomitant with or shortly followed (within a time window <500 ms in human) by the appearance of a spike-and-wave complex. The occurrence of such a cortical paroxysmal event will thus interfere with the post-stimulus neuronal activity (lasting ∼500 ms) required to allow the initial cortical sensory response to elicit a conscious experience [78]. Supporting this hypothesis, it has been shown that a direct electrical stimulation of the human primary somatosensory cortex precludes the conscious perception of an external event when the cortical stimulation is applied shortly after (<500 ms) the sensory stimulus [79].

Alternatively, it has been recently proposed that the loss of consciousness during absence seizures is caused by a disruption of the normal information processing in large-scale brain networks. Recent investigations in patients with generalized SWDs, associating EEG and fMRI analyses, indicated bilateral thalamic activation with variable fMRI cortical signals, decreasing [80] or increasing [81] in lateral frontal and parietal association areas. Combining these data with consistent results obtained in animal models [2], [4], [32] leads to the assumption that a transient functional disorder in bilateral association cortex and related subcortical structures is primarily responsible for the impairment of consciousness and the inability to generate a full-blown conscious sensation during absence seizures.

## References

[1] Panayiotopoulos CP, Koutroumanidis M, Giannakodimos S, Agathonikou A (1997) Idiopathic generalised epilepsy in adults manifested by phantom absences, generalised tonic-clonic seizures, and frequent absence status. J Neurol Neurosurg Psychiatry 63: 622–627. 9408104 10.1136/jnnp.63.5.622 PMC2169820

[2] Blumenfeld H (2005) Consciousness and epilepsy: why are patients with absence seizures absent? Prog Brain Res 150: 271–286. 16186030 10.1016/S0079-6123(05)50020-7 PMC3153469

[3] Panayiotopoulos CP (2008) Typical absence seizures and related epileptic syndromes: assessment of current state and directions for future research. Epilepsia 49: 2131–2139. 19049569 10.1111/j.1528-1167.2008.01777.x

[4] Cavanna AE, Monaco F (2009) Brain mechanisms of altered conscious states during epileptic seizures. Nat Rev Neurol 5: 267–276. 19488084 10.1038/nrneurol.2009.38

[5] Gibbs F, Davis H, Lennox WG (1935) The electroencephalogram in epilepsy and in conditions of impaired consciousness. Arch Neurol Psychiatr 34: 1133–1148.

[6] Williams D (1953) A study of thalamic and cortical rhythms in petit mal. Brain 76: 50–69. 13041922 10.1093/brain/76.1.50

[7] Berg AT, Berkovic SF, Brodie MJ, Buchhalter J, Cross JH, et al (2010) Revised terminology and concepts for organization of seizures and epilepsies: report of the ILAE Commission on Classification and Terminology, 2005–2009. Epilepsia 51: 676–685. 20196795 10.1111/j.1528-1167.2010.02522.x

[8] Lemieux J, Blume WT (1986) Topographical evolution of spike-wave complexes. Brain Res 373: 275–287. 3719312 10.1016/0006-8993(86)90342-2

[9] Blume W, Lemieux JF (1988) Morphology of spikes in spike-and-wave complexes. Electroencephalogr Clin Neurophysiol. 69: 508–515. 10.1016/0013-4694(88)90162-9 2453327

[10] Holmes MD, Brown M, Tucker DM (2004) Are “generalized” seizures truly generalized? Evidence of localized mesial frontal and frontopolar discharges in absence. Epilepsia 45: 1568–1579. 15571515 10.1111/j.0013-9580.2004.23204.x

[11] Sadleir LG, Farrell K, Smith S, Connolly MB, Scheffer IE (2006) Electroclinical features of absence seizures in childhood absence epilepsy. Neurology 67: 413–418. 16894100 10.1212/01.wnl.0000228257.60184.82

[12] Westmijse I, Ossenblok P, Gunning B, van Luijtelaar G (2009) Onset and propagation of spike and slow wave discharges in human absence epilepsy: A MEG study. Epilepsia 50: 2538–2548. 19519798 10.1111/j.1528-1167.2009.02162.x

[13] Danober L, Deransart C, Depaulis A, Vergnes M, Marescaux C (1998) Pathophysiological mechanisms of genetic absence epilepsy in the rat. Prog Neurobiol 55: 27–57. 9602499 10.1016/s0301-0082(97)00091-9

[14] Depaulis A, van Luijtelaar G (2006) Genetics models of Absence Epilepsy in the rat. In: Schwartzkroin PA, Pitkanen A, Moshé SL, editors. Models of Seizures and Epilepsy. London: Elsevier Academic Press. 233–248.

[15] Meeren HK, Pijn JP, Van Luijtelaar EL, Coenen AM, Lopes da Silva FH (2002) Cortical focus drives widespread corticothalamic networks during spontaneous absence seizures in rats. J Neurosci 22: 1480–1495. 11850474 10.1523/JNEUROSCI.22-04-01480.2002 PMC6757554

[16] Meeren H, van Luijtelaar G, Lopes da Silva F, Coenen A (2005) Evolving concepts on the pathophysiology of absence seizures: the cortical focus theory. Arch Neurol 62: 371–376. 15767501 10.1001/archneur.62.3.371

[17] Polack PO, Guillemain I, Hu E, Deransart C, Depaulis A, et al (2007) Deep layer somatosensory cortical neurons initiate spike-and-wave discharges in a genetic model of absence seizures. J Neurosci 27: 6590–6599. 17567820 10.1523/JNEUROSCI.0753-07.2007 PMC6672429

[18] David O, Guillemain I, Saillet S, Reyt S, Deransart C, et al (2008) Identifying neural drivers with functional MRI: an electrophysiological validation. PLoS Biol 6: 2683–2697. 19108604 10.1371/journal.pbio.0060315 PMC2605917

[19] Polack PO, Mahon S, Chavez M, Charpier S (2009) Inactivation of the somatosensory cortex prevents paroxysmal oscillations in cortical and related thalamic neurons in a genetic model of absence epilepsy. Cereb Cortex 19: 2078–2091. 19276326 10.1093/cercor/bhn237

[20] Chipaux M, Charpier S, Polack PO (2011) Chloride-mediated inhibition of the ictogenic neurones initiating genetically-determined absence seizures. Neuroscience 192: 642–651. 21704682 10.1016/j.neuroscience.2011.06.037

[21] Timofeev I, Steriade M (2004) Neocortical seizures: initiation, development and cessation. Neuroscience 123: 299–336. 14698741 10.1016/j.neuroscience.2003.08.051

[22] Paz J, Polack PO, Slaght SJ, Deniau JM, Chavez M, et al.. (2009) Cortical initiation of absence seizures, propagation to basal ganglia and back to the cortex. In: Tang FR, editor. Pan-brain abnormal neural network in epilepsy. Kerala: Research Signpost. 41–65.

[23] Schwab R (1939) A method of measuring consciousness in petit mal epilepsy. J Nerv Ment Dis 89: 690–691.

[24] Mirsky AF, Vanburen JM (1965) On the nature of the “absence” in centrencephalic epilepsy: a study of some behavioral electroencephalographic and autonomic factors. Electroencephalogr Clin Neurophysiol 18: 334–348. 14267826 10.1016/0013-4694(65)90053-2

[25] Kostopoulos GK (2001) Involvement of the thalamocortical system in epileptic loss of consciousness. Epilepsia 42 Suppl 3 13–19. 10.1046/j.1528-1157.2001.042suppl.3013.x 11520316

[26] Blumenfeld H, Taylor J (2003) Why do seizures cause loss of consciousness? Neuroscientist 9: 301–310. 14580115 10.1177/1073858403255624

[27] Jung R (1939) Uber vegetative Reaktionen und Hemmungswirkung von Sinnesreizen im kleinen epileptischen Anfall. Nervenarzt 12: 169–185.

[28] Fedio P, Mirsky AF (1969) Selective intellectual deficits in children with temporal lobe or centrencephalic epilepsy. Neuropsychologia 7: 287–300.

[29] Duncan C (1988) Application of event-related brain potentials to the analysis of interictal attention in absence epilepsy. In: Myslobodsky MS, Mirsky AF, editors. Elements of Petit Mal Epilepsy. New York: Peter Lang. 341–364.

[30] Duncan CC, Mirsky AF, Lovelace CT, Theodore WH (2009) Assessment of the attention impairment in absence epilepsy: comparison of visual and auditory P300. Int J Psychophysiol 73: 118–122. 19414047 10.1016/j.ijpsycho.2009.03.005 PMC2733346

[31] Orren MM (1978) Evoked potential studies in petit mal epilepsy. Visual information processing in relation to spike and wave discharges. Electroencephalogr Clin Neurophysiol Suppl 34: 251–257. 108074

[32] Blumenfeld H, Westerveld M, Ostroff RB, Vanderhill SD, Freeman J, et al (2003) Selective frontal, parietal, and temporal networks in generalized seizures. Neuroimage 19: 1556–1566. 12948711 10.1016/s1053-8119(03)00204-0

[33] van Luijtelaar G, Sitnikova E (2006) Global and focal aspects of absence epilepsy: the contribution of genetic models. Neurosci Biobehav Rev 30: 983–1003. 16725200 10.1016/j.neubiorev.2006.03.002

[34] Polack PO, Charpier S (2009) Ethosuximide converts ictogenic neurons initiating absence seizures into normal neurons in a genetic model. Epilepsia 50: 1816–1820. 19260940 10.1111/j.1528-1167.2009.02047.x

[35] Vergnes M, Marescaux C, Boehrer A, Depaulis A (1991) Are rats with genetic absence epilepsy behaviorally impaired? Epilepsy Res 9: 97–104. 1794357 10.1016/0920-1211(91)90019-c

[36] Getova D, Bowery NG, Spassov V (1997) Effects of GABAB receptor antagonists on learning and memory retention in a rat model of absence epilepsy. Eur J Pharmacol 320: 9–13. 9049596 10.1016/s0014-2999(96)00877-1

[37] Pastor MA, Valencia M, Artieda J, Alegre M, Masdeu JC (2007) Topography of cortical activation differs for fundamental and harmonic frequencies of the steady-state visual-evoked responses. An EEG and PET H215O study. Cereb Cortex 17: 1899–1905. 17060366 10.1093/cercor/bhl098

[38] Vialatte FB, Maurice M, Dauwels J, Cichocki A (2010) Steady-state visually evoked potentials: focus on essential paradigms and future perspectives. Prog Neurobiol 90: 418–438. 19963032 10.1016/j.pneurobio.2009.11.005

[39] Pidoux M, Mahon S, Deniau JM, Charpier S (2011) Integration and propagation of somatosensory responses in the corticostriatal pathway: an intracellular study in vivo. J Physiol 589: 263–281. 21059765 10.1113/jphysiol.2010.199646 PMC3043532

[40] Commission on classification and terminology of the ILAE (1989) Proposal for revised classification of epilepsies and epileptic syndromes. Commission on Classification and Terminology of the International League Against Epilepsy. Epilepsia 30: 389–399. 2502382 10.1111/j.1528-1157.1989.tb05316.x

[41] Hirsch E, Panayiotopoulos CP (2005) Epilepsie-absences de l’enfance et syndromes apparentés. In: Bureau M, Roger J, Dravet C, Genton P, Tassinari CA, et al.., editors. Les syndromes épileptiques de l’enfant et de l’adolescent. London: John Libbey. 315–336.

[42] Jasper H (1958) Report of the committee on methods of clinical examination in electroenchephalography. Electroencephalogr Clin Neurophysiol 10: 370–375.

[43] Rees G, Kreiman G, Koch C (2002) Neural correlates of consciousness in humans. Nat Rev Neurosci 3: 261–270. 11967556 10.1038/nrn783

[44] Bruno RM, Sakmann B (2006) Cortex is driven by weak but synchronously active thalamocortical synapses. Science 312: 1622–1627. 16778049 10.1126/science.1124593

[45] Paxinos G, Watson C (1986) The brain in stereotaxic coordinates. Sydney: Academic Press. 456 p.

[46] Chung S, Li X, Nelson SB (2002) Short-term depression at thalamocortical synapses contributes to rapid adaptation of cortical sensory responses in vivo. Neuron 34: 437–446. 11988174 10.1016/s0896-6273(02)00659-1

[47] Carvell GE, Simons DJ (1990) Biometric analyses of vibrissal tactile discrimination in the rat. J Neurosci 10: 2638–2648. 2388081 10.1523/JNEUROSCI.10-08-02638.1990 PMC6570272

[48] Fox K (2008) Barrel cortex. Cambridge: Cambridge University Press. 298 p.

[49] Mahon S, Deniau JM, Charpier S (2003) Various synaptic activities and firing patterns in cortico-striatal and striatal neurons in vivo. J Physiol Paris 97: 557–566. 15242665 10.1016/j.jphysparis.2004.01.013

[50] Mahon S, Charpier S (2012) Bidirectional plasticity of intrinsic excitability controls sensory inputs efficiency in layer 5 barrel cortex neurons in vivo. J Neurosci 32: 11377–11389. 22895720 10.1523/JNEUROSCI.0415-12.2012 PMC6621180

[51] Syed E, Sharott A, Moll CK, Engel AK, Kral A (2011) Effect of sensory stimulation in rat barrel cortex, dorsolateral striatum and on corticostriatal functional connectivity. Eur J Neurosci 33: 461–470. 21175884 10.1111/j.1460-9568.2010.07549.x

[52] Manning JP, Richards DA, Leresche N, Crunelli V, Bowery NG (2004) Cortical-area specific block of genetically determined absence seizures by ethosuximide. Neuroscience 123: 5–9. 14667436 10.1016/j.neuroscience.2003.09.026

[53] Gutnick M, Crill WE (1995) The cortical neuron as an electrophysiological unit. In Gutnick MJ, Mody I editors. The Cortical Neuron. Oxford: Oxford University Press. 33–52.

[54] Nicolelis MA, Fanselow EE (2002) Thalamocortical optimization of tactile processing according to behavioral state. Nat Neurosci 5: 517–523. 12037519 10.1038/nn0602-517

[55] Conte B, Cutrufo C, Manzini S (1996) Electrocorticographic desynchronization after application of visceral and somatic noxious stimuli in urethane-anesthetized rats: effect of intrathecal administration of tachykinin (NK 1 or NK 2) receptor antagonists. J Pharmacol Exp Ther 276: 212–218. 8558433

[56] Simons D, Carvell GE, Hershey AE, Bryant DP (1992) Responses of barrel cortex neurons in awake rats and effects of urethane anesthesia. Exp Brain Res 91: 259–272. 1459228 10.1007/BF00231659

[57] Steriade M, McCormick DA, Sejnowski TJ (1993) Thalamocortical oscillations in the sleeping and aroused brain. Science 262: 679–685. 8235588 10.1126/science.8235588

[58] Steriade M (2000) Corticothalamic resonance, states of vigilance and mentation. Neuroscience 101: 243–276. 11074149 10.1016/s0306-4522(00)00353-5

[59] Llinas RR, Steriade M (2006) Bursting of thalamic neurons and states of vigilance. J Neurophysiol 95: 3297–3308. 16554502 10.1152/jn.00166.2006

[60] Pinault D, Leresche N, Charpier S, Deniau JM, Marescaux C, et al (1998) Intracellular recordings in thalamic neurones during spontaneous spike and wave discharges in rats with absence epilepsy. J Physiol 509: 449–456. 9575294 10.1111/j.1469-7793.1998.449bn.x PMC2230966

[61] Slaght SJ, Paz T, Mahon S, Maurice N, Charpier S, et al (2002) Functional organization of the circuits connecting the cerebral cortex and the basal ganglia: implications for the role of the basal ganglia in epilepsy. Epileptic Disord 4 Suppl 3 S9–22. 12495871

[62] Pinault D (2003) Cellular interactions in the rat somatosensory thalamocortical system during normal and epileptic 5–9 Hz oscillations. J Physiol 552: 881–905. 12923213 10.1113/jphysiol.2003.046573 PMC2343451

[63] Brecht M, Sakmann B (2002) Whisker maps of neuronal subclasses of the rat ventral posterior medial thalamus, identified by whole-cell voltage recording and morphological reconstruction. J Physiol 538: 495–515. 11790815 10.1113/jphysiol.2001.012334 PMC2290066

[64] Wiest M, Nicolelis MA (2003) Behavioral detection of tactile stimuli during 7–12 Hz cortical oscillations in awake rats. Nat Neurosci 6: 913–914. 12897789 10.1038/nn1107

[65] Shaw F (2004) Is spontaneous high-voltage rhythmic spike discharge in Long Evans rats an absence-like seizure activity? J Neurophysiol 9: 63–77. 10.1152/jn.00487.2003 12826656

[66] Polack PO, Charpier S (2006) Intracellular activity of cortical and thalamic neurones during high-voltage rhythmic spike discharge in Long-Evans rats in vivo. J Physiol 571: 461–476. 16410284 10.1113/jphysiol.2005.100925 PMC1796797

[67] Charpier S, Leresche N, Deniau JM, Mahon S, Hughes SW, et al (1999) On the putative contribution of GABA(B) receptors to the electrical events occurring during spontaneous spike and wave discharges. Neuropharmacology 38: 1699–1706. 10587086 10.1016/s0028-3908(99)00139-2

[68] Azouz R, Gray CM (1999) Cellular mechanisms contributing to response variability of cortical neurons in vivo. J Neurosci 19: 2209–2223. 10066274 10.1523/JNEUROSCI.19-06-02209.1999 PMC6782570

[69] Steriade M, Dossi RC, Nuñez A (1991) Network modulation of a slow intrinsic oscillation of cat thalamocortical neurons implicated in sleep delta waves: cortically induced synchronization and brainstem cholinergic suppression. J Neurosci 11: 3200–3217. 1941080 10.1523/JNEUROSCI.11-10-03200.1991 PMC6575431

[70] Young G, Pigott SE (1999) Neurobiological basis of consciousness. Arch Neurol 56: 153–157. 10025420 10.1001/archneur.56.2.153

[71] Edeline J (2003) The thalamo-cortical auditory receptive fields: Regulation by the states of vigilance, learning and the neuromodulatory systems. Exp Brain Res 153: 554–572. 14517594 10.1007/s00221-003-1608-0

[72] Bastuji H, García-Larrea L (1999) Evoked potentials as a tool for the investigation of human sleep. Sleep Med Rev 3: 23–45. 15310488 10.1016/s1087-0792(99)90012-6

[73] Portas C, Krakow K, Allen P, Josephs O, Armony JL, et al (2000) Auditory processing across the sleep-wake cycle: Simultaneous EEG and fMRI monitoring in humans. Neuron 28: 991–999. 11163282 10.1016/s0896-6273(00)00169-0

[74] Wygnanski-Jaffe T, Panton CM, Buncic JR, Westall CA (2009) Paradoxical robust visual evoked potentials in young patients with cortical blindness. Doc Ophthalmol 119: 101–107. 19548015 10.1007/s10633-009-9176-7

[75] Dang-Vu TT, Bonjean M, Schabus M, Boly M, Darsaud A, et al (2011) Interplay between spontaneous and induced brain activity during human non-rapid eye movement sleep. Proc Natl Acad Sci USA 108: 15438–15443. 21896732 10.1073/pnas.1112503108 PMC3174676

[76] Schabus M, Dang-Vu TT, Heib DP, Boly M, Desseilles M, et al (2012) The Fate of Incoming Stimuli during NREM Sleep is Determined by Spindles and the Phase of the Slow Oscillation. Front Neurol 3: 40. 22493589 10.3389/fneur.2012.00040 PMC3319907

[77] Massimini M, Rosanova M, Mariotti M (2003) EEG slow (approximately 1 Hz) waves are associated with non stationarity of thalamocortical sensory processing in the sleeping human. J Neurophysiol 89: 1205–1213. 12626608 10.1152/jn.00373.2002

[78] Libet B, Alberts WW, Wright EW Jr, Feinstein B (1967) Responses of human somatosensory cortex to stimuli below threshold for conscious sensation. Science 158: 1597–1600. 6060369 10.1126/science.158.3808.1597

[79] Libet B, Alberts WW, Wright EW Jr, Feinstein B (1972) Cortical and thalamic activation in conscious sensory experience. In: Somjen GG, editor. Neurophysiology studied in man. Amsterdam: Excerpta Medica. 157–168.

[80] Hamandi K, Salek-Haddadi A, Laufs H, Liston A, Friston K, et al (2006) EEG-fMRI of idiopathic and secondarily generalized epilepsies. Neuroimage 31: 1700–1710. 16624589 10.1016/j.neuroimage.2006.02.016

[81] Blumenfeld H, Varghese GI, Purcaro MJ, Motelow JE, Enev M, et al (2009) Cortical and subcortical networks in human secondarily generalized tonic-clonic seizures. Brain 132: 999–1012. 19339252 10.1093/brain/awp028 PMC2724910

